# A Novel SARS-CoV-2 Viral Sequence Bioinformatic Pipeline Has Found Genetic Evidence That the Viral 3′ Untranslated Region (UTR) Is Evolving and Generating Increased Viral Diversity

**DOI:** 10.3389/fmicb.2021.665041

**Published:** 2021-06-21

**Authors:** Carlos Farkas, Andy Mella, Maxime Turgeon, Jody J. Haigh

**Affiliations:** ^1^Research Institute in Oncology and Hematology (RIOH), CancerCare Manitoba, Winnipeg, MB, Canada; ^2^Department of Pharmacology and Therapeutics, Rady Faculty of Health Sciences, University of Manitoba, Winnipeg, MB, Canada; ^3^Departamento de Física, Facultad de Ciencias Físicas y Matemáticas, Universidad de Chile, Santiago, Chile; ^4^Instituto de Ciencias Naturales, Universidad de las Américas, Santiago, Chile; ^5^Department of Statistics, University of Manitoba, Winnipeg, MB, Canada; ^6^Department of Computer Science, University of Manitoba, Winnipeg, MB, Canada

**Keywords:** 3cpsdummy′UTR, SARS-CoV-2 variants, nucleotide diversity (π), Tajima’s D-statistic, viral evolution, VCF

## Abstract

An unprecedented amount of SARS-CoV-2 sequencing has been performed, however, novel bioinformatic tools to cope with and process these large datasets is needed. Here, we have devised a bioinformatic pipeline that inputs SARS-CoV-2 genome sequencing in FASTA/FASTQ format and outputs a single Variant Calling Format file that can be processed to obtain variant annotations and perform downstream population genetic testing. As proof of concept, we have analyzed over 229,000 SARS-CoV-2 viral sequences up until November 30, 2020. We have identified over 39,000 variants worldwide with increased polymorphisms, spanning the ORF3a gene as well as the 3′ untranslated (UTR) regions, specifically in the conserved stem loop region of SARS-CoV-2 which is accumulating greater observed viral diversity relative to chance variation. Our analysis pipeline has also discovered the existence of SARS-CoV-2 hypermutation with low frequency (less than in 2% of genomes) likely arising through host immune responses and not due to sequencing errors. Among annotated non-sense variants with a population frequency over 1%, recurrent inactivation of the ORF8 gene was found. This was found to be present in the newly identified B.1.1.7 SARS-CoV-2 lineage that originated in the United Kingdom. Almost all VOC-containing genomes possess one stop codon in ORF8 gene (Q27^∗^), however, 13% of these genomes also contains another stop codon (K68^∗^), suggesting that ORF8 loss does not interfere with SARS-CoV-2 spread and may play a role in its increased virulence. We have developed this computational pipeline to assist researchers in the rapid analysis and characterization of SARS-CoV-2 variation.

## Introduction

The novel SARS-CoV-2 coronavirus that causes COVID-19 has surpassed 95 million infections worldwide within 1 year of pandemic, resulting in more than two million deaths until January 2021^1^ (Dong et al., 2020). In-depth characterization of this virus is urgently needed to improve outbreak surveillance, vaccine development and for effective treatments now and in the immediate future. SARS-CoV-2 is a positive single-stranded RNA virus (+ssRNA) with a crown-like appearance observed by electron microscopy that is due to the presence of spike glycoproteins on the lipid bilayer envelope (Cui et al., 2019; Ke et al., 2020). Another two transmembrane proteins are incorporated into the envelope: small envelope protein (E) and membrane protein (M) (Wu et al., 2020). As seen with SARS-CoV-1, SARS-CoV-2 binds through its Spike glycoprotein to cell membrane-bound angiotensin-converting enzyme 2 (ACE2) for entry into host cells (Crackower et al., 2002; Li et al., 2003; Ge et al., 2013; Hoffmann et al., 2020). Due to the importance of this Spike protein in SARS-CoV-2 infection, variants occurring in this protein are critical, since some can confer improved fitness to SARS-CoV-2 (Starr et al., 2020) and others affect antigenicity and maybe affect vaccine efficiency (Li J. et al., 2020). SARS-CoV-2 sequencing has been standardized through initiatives such as the Advancing Real-Time Infection Control Network (ARTIC) international initiative (Tyson et al., 2020) in which Illumina (Hourdel et al., 2020) or Oxford Nanopore (Freed et al., 2020; Li Q. et al., 2020) sequencing is carried out prior to whole viral genome amplification by tiling PCR or metagenomic approaches. The Centers for Disease Control and Prevention (CDC) maintains a GitHub page^2^ detailing recommended protocols, tools and resources for SARS-CoV-2 whole genome sequencing on the mentioned two sequencing platforms, including, PacBio and Ion Torrent technologies. After sequencing, initiatives such as GISAID^3^ (Elbe and Buckland-Merrett, 2017; Shu and McCauley, 2017) and the Sequence Read Archive (SRA^4^) have been storing SARS-CoV-2 sequencing datasets worldwide from the beginning of the pandemic starting in January 2020, allowing researchers to track fixed variants and follow viral evolution by geographical region. The unprecedented amount of SARS-CoV-2 whole genome sequencing data can help to (1) characterize viral variants that occur within a given host, (2) understand variant fixation in a given population, and (3) understand how the virus changes over time. In fact, the Spike protein mutation D614G global transmission was discovered in this way and is associated with higher viral titers and increased fitness (Korber et al., 2020; Plante et al., 2020).

The SARS-CoV-2 genome possess coding capacity for structural proteins and a variety of accessory Open Reading Frames (ORFs), assessed by both computationally predictions and ribosomal profiling techniques. The transcription of SARS-CoV-2 is constant from 5′UTR toward ORF1a and ORF1b structural proteins, and steadily increase toward the 3′ end due to the nested transcription of sub-genomic viral RNAs (Finkel et al., 2021). As a consequence of increased transcription, novel overlapping ORFs can be readily found in SARS-CoV-2 (Nelson et al., 2020; Finkel et al., 2021) including in-frame fusions (Nomburg et al., 2020). These accessory ORFs has been demonstrated to play a role in modulating the immune response from the host (Lei et al., 2020) and can disrupt host cell signaling capacity suppressing STAT1/2 phosphorylation, inhibiting interferon gamma mediated response, and causing immune evasion (Xia et al., 2020). Population-fixed variants can disrupt these ORFs by creating new stop codons, a phenomenon already demonstrated for ORF3a (Lam et al., 2020), ORF6 (Queromes et al., 2021) and ORF8 (Gong et al., 2020; Ngernmuen et al., 2020; Flower et al., 2021) amongst other ORFs. Also, variants toward the 3′UTR of the virus can confer resistance to host miRNA viral targeting since several human miRNAs are predicted to prevent virus replication by binding to this untranslated region (Chen and Zhong, 2020; Mukherjee and Goswami, 2020). Thus, it is useful to track these newly fixed viral variants over time across populations using effective bioinformatic tools that are appropriate for these tasks.

Several bioinformatic pipelines have been developed to assist in the genomic epidemiology of SARS-CoV-2 that output sequence alignment analysis and/or variants in various formats. Tools such as VIRULIGN (Libin et al., 2019) and ViralMSA (Moshiri, 2020) rely on multiple sequence alignment algorithms to assess identity and further annotation of sequences by outputting viral sequence alignments. Similarly, the pangolin pipeline efficiently assigns input viral sequences to SARS-CoV-2 lineages by using sequence alignment and phylogenetic identification and has the potential to infer variants specifically associated to a specific lineage (Rambaut et al., 2020). Also tools such as CorGAT, can assist in the functional annotation of SARS-CoV-2 genomes by sequence alignment and outputting a pseudo-VCF file containing detected variants (Chiara et al., 2020). Clearly, multiple sequence alignment tools are useful in terms of phylogenetic reconstruction and identification, but the process to convert FASTA alignments to variant calls could be ambiguous depending on the variant report format, thus a uniform variant output format such as the Variant Calling Format (VCF) is convenient and suitable for downstream genetic analyses (Danecek et al., 2011). For these reasons, we devised a pipeline that can input viral Next Generation Sequencing (NGS) datasets or FASTA SARS-CoV-2 genome sequences and process them to obtain aggregated variants in standard population-aware VCF format, an output format that is suitable for variant filtering, annotation and calculation of nucleotide diversity and/or Tajima’s D parameters, among other applications.

## Methods

### Data and Code Availability

17,560 sequencing datasets were downloaded from Sequence Read Archive Repository (SRA^5^) from December 1, 2019 until July 28, 2020. Associated sequencing run accessions, sequencing metadata and related BioProjects are listed in Supplementary Table 1. 229,124 FASTA genomes and associated sequencing metadata were downloaded from GISAID database from January 1, 2019 until November 30, 2020, specifying “human” as source host^6^. Associated metadata and acknowledgments to laboratories/consortia involved in the corresponding genome sequencing is listed in Supplementary Tables 2, 3, respectively. Aggregated variants in VCF format for the latter genomes including the associated predictions by SnpEff program (Cingolani et al., 2012) are available here: https://usegalaxy.org/u/carlosfarkas/h/snpeffsars-cov-2. 36,308 GISAID FASTA sequences from lineage B.1.1.7 were downloaded from GISAID database from January 1, 2019 until January 27, 2021, specifying “human” as source host and “B.1.1.7” as lineage in GISAID database. Aggregated variants in VCF format for the latter genomes including the associated sequencing metadata and acknowledgments are available here: https://usegalaxy.org/u/carlosfarkas/h/b117. The code generated during this study to replicate most of the computational calculations performed in this manuscript is available at the following GitHub repository: https://github.com/cfarkas/SARS-CoV-2-freebayes.

### Next-Generation Sequencing and FASTA Dataset Processing

**FIGURE 1:**
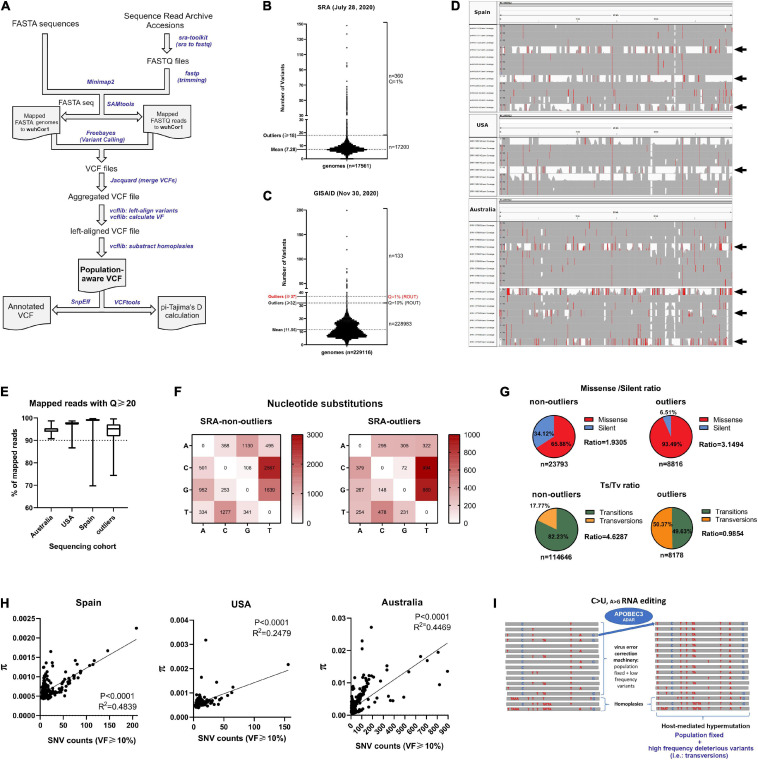
SARS-CoV-2 genome analysis reveals inter-host diversity that in extreme cases leads to hypermutation. **(A)** Pipeline overview to process GISAID FASTA genome sequences and Sequence Read Archive datasets. Both inputs are aligned to SARS-CoV-2 reference genome (wuhCor1) and variant calling is performed to obtain a single population-aware VCF file suitable for downstream genetic analysis. Streamlined bioinformatic tools are depicted with blue letters. **(B)** Major viral frequency variants (via a consensus calling approach) for 17,560 next generation sequencing (NGS) datasets downloaded from SRA, separated by non-outliers (*n* = 17,200) and outliers (*n* = 360, *Q* = 1%, Grubbs’s test). Outlier number and mean of variants are depicted at left. **(C)** Same as B for 229,124 SARS-CoV-2 GISAID genomes, separated by non-outliers (*n* = 228,093) and outliers (*n* = 143, *Q* = 10%, Grubbs’s test). Outlier number and mean of variants are depicted at left. **(D)** IGV snapshots of outliers and non-outlier NGS samples from C. Outlier samples are depicted with black arrows, exceeding number of variants from non-outliers. Single nucleotide polymorphisms are depicted in red if nucleotide differs from the reference sequence by greater or equal to 50% of quality weighted reads. **(E)** Q20 statistics obtained with SeqKit program for mapped reads of 374 NGS datasets from Spain, 215 NGS datasets from United States, 397 NGS datasets from Australia and 360 outlier NGS datasets from SRA. Percentages are depicted in the *y*-axis. **(F)** Nucleotide change frequencies from 17,200 SRA NGS aggregated variants (non-outliers, left) and from 360 aggregated outlier variants (right), both annotated with SnpEff program. Frequency boxes are colored from white to dark red as number of changes increases. **(G)** (Upper) Pie charts depicting missense/silent ratios registered in the non-outliers and outlier NGS samples. Values are denoted as percentages and the total number of variants are denoted in the bottom of the graphs. (Lower) Same as upper, for transitions/transversions ratios (Ts/Tv). **(H)** Correlation between Average nucleotide diversity (π) provided by inStrain program and SNV counts (VF > 10%) for Spain (*n* = 374, left), United States (*n* = 215, middle) and Australian NGS samples (*n* = 397, right). In the three countries, the two variables tend to increase together (see *r*-values of Spearman correlation analyses). **(I)** Proposed model of how APOBEC3G and ADAR complex (with minor contributions) can lead to hypermutation of SARS-CoV-2 (C > U and A > G editing) accompanied by intra-host diversity, homoplasies and increased transversions. In the majority of infections, it is probable that micro diversity is maintained at low frequencies due the action of the virus error correction machinery.

To process next generation sequencing datasets, we employed our pipeline (SARS-CoV-2_freebayes) consisting of a bash/UNIX script that runs several programs in sequential order. We processed imputed list of SRA accessions with sra-tools^7^, generating compressed FASTQ files per sequencing, automatically trimmed with fastp tool (Chen et al., 2018). Then, we aligned each trimmed fastq file against a provided reference genome (Wuhan-Hu-1, GenBank Accession: MN908947.3) using Minimap2 splice-aware aligner in preset mode -ax sr (Li, 2018). We sorted and indexed the resulting BAM files by using Samtools (Li et al., 2009) and performed variant calling on every sorted BAM file, obtaining major frequency viral variants per genome in VCF format using the Freebayes variant calling program, as frequency-based pooled caller (−F 0.49)^8^ (Garrison and Marth, 2012). Then, we used Jacquard program^9^ in the python environment (Sanner, 1999) to merge every VCF file containing variants associated to each bam file into a single VCF file, containing aggregated variants from all genomes. In the resulting merged VCF file, we recalculated viral frequencies using several UNIX tools (Kernighan and Morgan, 1982), in combination with vcflib^10^. We used the variants per genome logfile “logfile_variants_SRA_freebayes” to construct Figure 1B using GraphPad Prism 8 software^11^. We processed GISAID FASTA genomes in a similar manner. We preprocess a single GISAID genome collection with SeqKit (Shen et al., 2016) to decompose a single FASTA file into individual FASTA files, each file containing a single genome. Then, we aligned every FASTA genome against SARS-CoV-2 reference genome (NC_045512.2) using Minimap2 aligner with preset -ax asm5 (Li, 2018) and performed variant calling on each BAM file using Freebayes variant caller with –min-alternate-count 1 (C 1) option (see text footnote 8), outputting variants in VCF format. With these operations, we obtained major frequency viral variants in VCF format from each FASTA genome. Then, we aggregated variants into a single VCF file, as described with Jacquard. We constructed Figure 1B graph by using variants per genome logfile, reported in the output file “logfile_variants_GISAID_freebayes” and imputed into the GraphPad Prism 8 software. We filtered out highly homoplasic sites from merged variant calls, as already reported to be frequent in SARS-CoV-2 sequencing see: https://virological.org/t/issues-with-sars-cov-2-sequencing-data/473. All these computational analyses are described here: https://github.com/cfarkas/SARS-CoV-2-freebayes (case examples I and II, respectively).

### Variant Visualization

We used the Integrative Genomics Viewer (IGV) software^12^ to visualize next generation sequencing alignments in bam format (Robinson et al., 2011, 2017; Thorvaldsdottir et al., 2013). To visualize major viral frequency variants, the variant frequency threshold was set at 0.49.

### SnpEff Annotation

**FIGURE 2:**
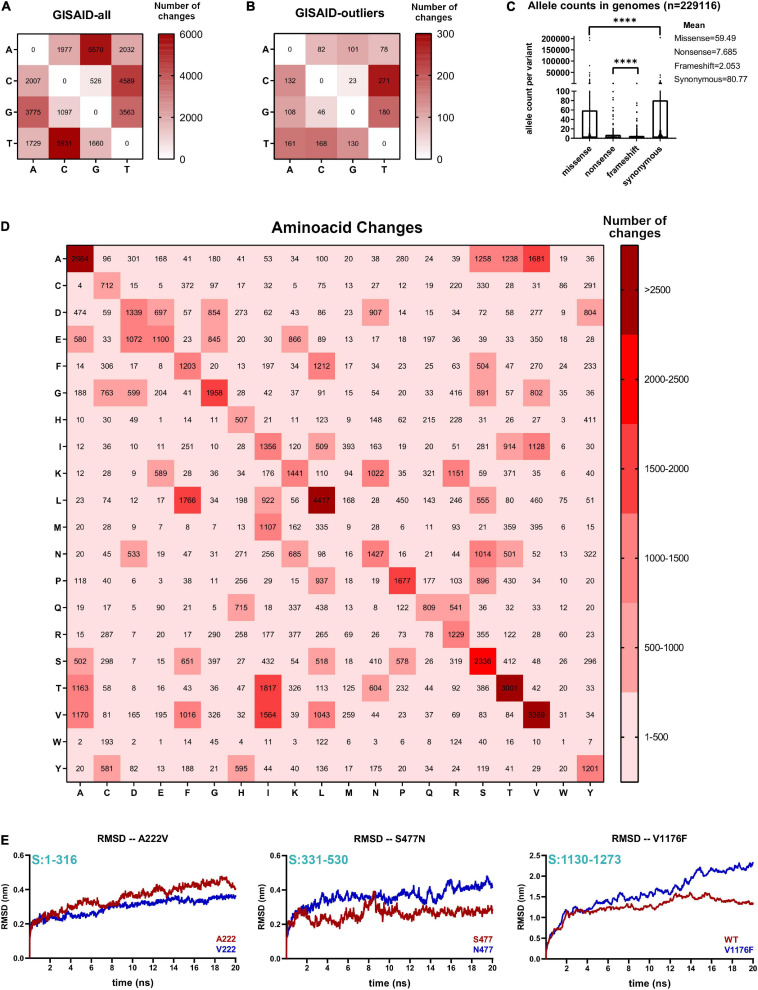
Non-neutral codon changes actively shape evolution of several SARS-CoV-2 proteins. **(A)** Nucleotide change frequencies from 229,124 aggregated GISAD genome variants annotated with SnpEff program. Frequency boxes are colored from white to dark red as number of changes increase. **(B)** Same as A for 133 aggregated GISAID genomes, corresponding to GISAID outlier samples. Frequencies boxes are colored from white to dark red, as the number of changes increases. **(C)** Missense, non-sense, frameshift, and synonymous number of occurrences in 229,124 GISAID genomes. Significance of comparisons were assessed with Mann-Whitney test (****P < 0.0001, ^*ns*^*P* > 0.05). **(D)** Plot of change frequencies across 20 amino acid changes in SARS-CoV-2. Changes are grouped in six categories and colored from light to dark red, according to the number of changes. **(E)** Spike protein mutant molecular dynamics. (Left) RMSD values (in nanometers, nm) of 20 ns of simulation of the wild-type N-terminal domain (A222) or mutant domain (V222). Residue positions of the domain respect to the full Spike protein is depicted in light blue. (Middle) Same as left for the wild-type RBD domain of the Spike protein (S477) or mutant RBD domain (N477). Residue positions of the domain with respect to the full Spike protein that is depicted in light blue. (Right) Same as left for the wild-type stalk domain trimmer (V1176) or the mutant domain containing Phenylalanine in position 1176 (F1176).

We annotated merged variants from GISAID genomes (*n* = 229,124) using a repurposed version of SnpEff program, available in the Galaxy server (Giardine et al., 2005; Cingolani et al., 2012; Afgan et al., 2018). We parsed the resulting annotated VCF file using the SnpEff_processing.sh script, available here: github.com/cfarkas/SARS-CoV-2-freebayes/blob/master/SnpEff_processing.sh. Aminoacid change chart related from Figure 2D is available as SnpEff HTML output here: https://usegalaxy.org/u/carlosfarkas/h/snpeffsars-cov-2. All these computational analyses are described here: https://github.com/cfarkas/SARS-CoV-2-freebayes (case example III).

### Tajima’s D and Nucleotide Diversity (π) Calculation

We estimated Tajima’s D and nucleotide diversity (π) metrics by using vcftools program (Danecek et al., 2011) on every geographical region as follows: joint variant calls from 4,301, 12,000, 145,888, 47,683, 2,325 and 17211 GISAID FASTA genomes from Africa, Asia, Europe, North America, South America, and Oceania, respectively, were processed from the alignment to the variant calling step as described in “GISAID FASTA dataset processing section.” Then, we imputed merged variants from every geographical region into vcftools, specifying the –haploid flag and setting a genome wide scan of 50 bp in length. We merged bins containing non missing values of Tajima’s D and π into a single file and we further processed this file with our pi-tajima.sh script (available in our repository) to obtain bins with Tajima’s D values outside 95% CI. All these computational analyses are described here: https://github.com/cfarkas/SARS-CoV-2-freebayes (case example IV).

### Intra-Host Diversity and Low Frequency Viral Variants

We estimated nucleotide diversity in 397, 448 and 308 next generation sequencing (NGS) samples from Australia, Spain, and United States populations, respectively, by using aligned reads per sample in BAM format against SARS-CoV-2 reference genome. These BAM files were imputed in loop to InStrain program^13^ (Olm et al., 2020, 2021), obtaining several outputs such as analysis of coverage, intra-host diversity, SNV linkage, and sensitive SNP detection. As recommended by inStrain, we analyzed only sequencing samples with sufficient breadth of coverage (>0.9), resulting in 397, 374 and 216 NGS samples from Australia, Spain and from United States, respectively. The list of the NGS samples in the three populations, including the referred calculations, are detailed in the spreadsheet inStrain_results.xlsx, available here: https://github.com/cfarkas/SARS-CoV-2-freebayes. We correlated in each country the number of variants with viral frequency >5% against the nucleotide diversity (π) by using Spearman correlation. Spearman’s correlation coefficients (r) and confident *p*-values (P, to discard random sampling) were calculated in GraphPad Prism 8. The significance thresholds were as follows: *P* < 0.05^∗^, *P* < 0.01^∗∗^, *P* < 0.001^∗∗∗^, *P* < 0.0001^****^, *P* > 0.05 ns.

### Molecular Dynamics Simulations

We conducted molecular dynamics simulations of variants A222V (N-terminal of SARS-CoV-2 residues 1–316), S477N (RBM domain, residues 331–530) and V11766F (stalk domain trimmer, residues 1,130–1,273). The full Spike protein trimmer was obtained from I-TASSER and variants were modeled by using Foldx5, as previously described in the Free energy estimation calculations section (–command = BuildModel, first outputted model). We simulated wild-type and mutants structures to molecular dynamics by using GROMACS/2020.3 version, in gpu mode^14^ (Van Der Spoel et al., 2005; Kutzner et al., 2015).

**FIGURE 3:**
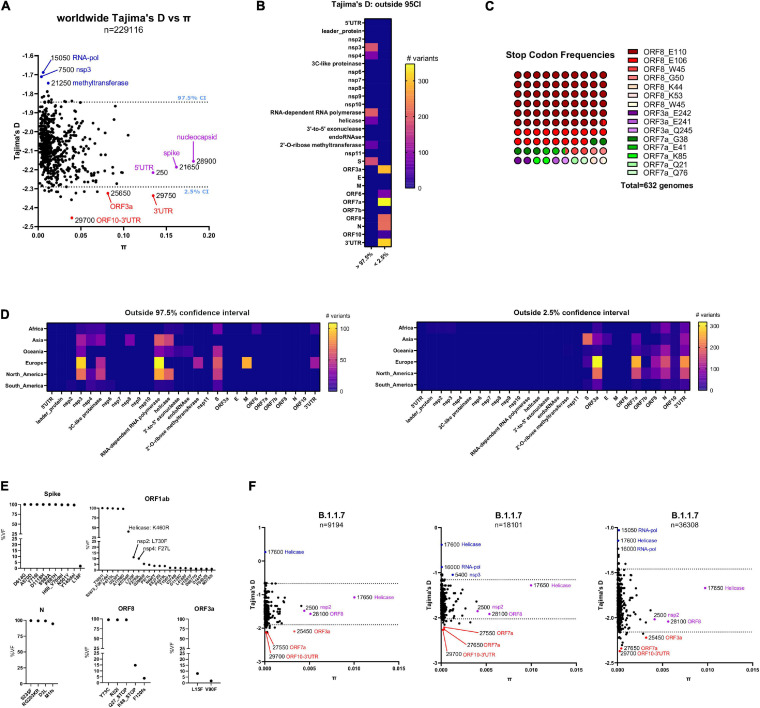
**(A)** Worldwide distribution of Tajima’s D values versus nucleotide diversity (π) for 595 bins of 50 bp in length derived from 229,124 GISAID genomes. The dashed lines correspond to the upper (97.5%) and lower (2.5%) percentiles of the empirical distribution of Tajima’s D for each bin. Genes containing the top three most extreme outlier bins are depicted in blue (in the upper 97.5th percentile) and red (in the lower 2.5th percentile). Also, genes containing the top three most diverse bins are depicted in purple. **(B)** Number of variants per gene derived from bins in the 97.5th and 2.5th percentile of the empirical distribution of Tajima’s D values from A, respectively. **(C)** Parts-of-whole plot of non-sense (Stop Codon) frequencies with worldwide viral frequencies over or equal to 1% from 229,124 GISAID genomes until November 30, 2020. ORF8 stop codons are depicted in a range of red colors, ORF3a stop codons are depicted with a range of purple colors, and ORF7a stop codons are depicted with a range of green colors. **(D)** Variants per gene derived from bins in the 2.5th and 97.5th percentile of the empirical distribution of Tajima’s D values, derived from African (*n* = 4301), Asian (*n* = 11,986), Oceanic (*n* = 17,211), North American (*n* = 47,658), South American (*n* = 2325), and European (*n* = 145,884). GISAID genomes, respectively. (Left) Plot of genes containing bins in the 97.5th percentile of the empirical distribution of Tajima’s D from GISAID genomes in every geographical region, until November 30, 2020. (Right) Plot of genes containing bins in the 2.5th percentile of the empirical distribution of Tajima’s D from GISAID genomes in every geographical region, until November 30, 2020. **(E)** Viral frequencies (as percentages) arranged per gene from variants contained in the VOC genomes submitted in GISAID until January 17th, 2021. **(F)** Same plot as **(A)** for GISAID genomes from the B.1.1.7 lineage submitted in GISAID until January 07, 2021 (left, 9194 genomes), January 17, 2021 (middle, 18,101 genomes), and January 27, 2020 (right, 36,308 genomes).

The xvg file records per picosecond were used to plot graphs from Figure 3C, on GraphPad Prism 8 software. PDB, solvated molecules (.gro) and correspondent compressed gromacs trajectories (with or without periodic border conditions) are available here : https://usegalaxy.org/u/carlosfarkas/h/sars-cov-2-proteins-and-trayectories. Detailed commands to obtain these trajectories are available in Supplementary File 1.

### Statistical Analysis

All statistical analyses were carried out by using GraphPad Prism 8 software (see text footnote 11). A Mann-Whitney test was used to account for the non-normality of the data. The significance thresholds were the following: *P* < 0.05^∗^, *P* < 0.01^∗∗^, *P* < 0.001^∗∗∗^, *P* > 0.05 ns. We interpreted the Spearman non-parametric correlation analyses as follows: perfect correlation (Dong et al., 2020), the two variables tend to increase or decrease together (0–1), the two variables do not vary together at all (0), one variable increase as the other decreases (−1–0), and perfect inverse correlations (−1). Since all correlations were calculated using more than 17 observations, *p*-values were computed using a normal approximation. We employed robust regression and outlier removal (ROUT) method (Motulsky and Brown, 2006) to remove outliers from data, with a strict false discovery ratio (Q = 10 and 1%).

## Results

### SARS-CoV-2 Genome Analysis Reveals Inter-Host Diversity That in Extreme Cases Leads to Hypermutation

To determine the degree of inter-host viral variation worldwide, we downloaded and analyzed 17,560 next-generation sequencing datasets from Sequence Read Archive (SRA) submitted since the beginning of the pandemic until July 28, 2020 (Supplementary Table 1) and 229,124 SARS-CoV-2 genome sequences available in the GISAID database up until November 30, 2020 (Supplementary Tables 2, 3, respectively). We inputted both datasets in out pipeline to obtain a single population-aware VCF file for subsequent genetic analyses. Variant calls were performed on each individual dataset and all resulting individual VCF files were merged, obtaining a single population-aware VCF file with calculated viral frequencies (see pipeline scheme in Figure 1A). Also, we accounted for sequencing artifacts and known homoplasies occurring in SARS-CoV-2 genomes due errors in sequencing and/or adaptor contamination were subtracted from these calls, as described here: https://virological.org/t/issues-with-sars-cov-2-sequencing-data/473. We benchmarked our pipeline with the pangolin pipeline for lineage reconstruction and Single Nucleotide Polymorphisms (SNP) detection, revealing good agreement on SNP detection between both (>95%), but also pipeline also accounted for the detection of Multi-Nucleotide polymorphisms (MNPs), Insertions/deletions and complex variants as well (Supplementary Figure 1). SARS-CoV-2 genomes from GISAID accounted for the presence of major viral frequency variants (via a consensus calling approach) compared to the Wuhan-Hu-1 genome assembly (wuhCor1) and the next-generation sequencing datasets (NGS) also allowed us to analyze intra-host diversity given the depth of sequencing. Until July 28, 2020, NGS datasets contained on average 7–8 viral variants with major alleles per genome (viral frequency > 0.5) (see “mean” in Figure 1B). As expected, GISAID datasets until November 30, 2020 contained more variants per genome on average because these sequences span more time and more variants and were fixed in SARS-CoV-2 over time [(Hourdel et al., 2020; Tyson et al., 2020) variants per genome, see “mean” in Figure 1C]. The distribution from both sources also identified outliers with more than 18 viral variants per genome in NGS samples and more than 37 variants per genome in GISAID FASTA genomes (2 and 0.05% in SRA and GIDAID sequencing datasets, see “outliers” in Figures 1B,C, respectively, Q = 1 and 10%, Grubbs’s test). Integrative genomics viewer (IGV) snapshots of outlier samples from Spain, United States and Australian sequencing datasets clearly show hypermutability to varying degrees (viral frequency > 0.49, see samples with black arrows, Figure 1D). Australian outlier samples represent an extreme case of hypermutability (see Figure 1D, bottom). Over 90% of the mapped reads against SARS-CoV-2 genome from the latter NGS datasets, including the outliers, contained phred quality scores of Q20 (99% base call accuracy), ensuring that the variant calling was reliable on these datasets and the variations registered are not due to sequencing errors (see Figure 1E). 16,307 aggregated variants from SRA datasets reflect that the most recurrent single nucleotide substitutions occurring in all genomes from SRA repository are enriched in C > U (C > T) transitions and G > U (G > T) transversions, changes already reported for SARS-CoV-2 and MERS-CoV genomes (Simmonds, 2020). As previously described, the C > U (C > T) changes are likely elicited by APOBEC deaminases (Di Giorgio et al., 2020; Figure 1F, left). This observation also applies for genomes containing an outlier number of variants from SRA, with the exception of A > G transitions, which are caused by the ADAR editing enzyme (Mourier et al., 2020; Figure 1F, right). Strikingly, most of the nucleotide substitutions harboring outlier samples from Figure 1C correspond to missense/non-sense variants rather than silent variants that are enriched in transversion changes, since in outlier samples the raw Transition/Transversion (Ts/Tv) ratio is near one and in non-outlier samples is 4.6 (Figure 1G). The amount of observed transversions in outlier samples correlates with the missense/non-sense vs. silent ratio observed in outliers, since transversions in viruses cause more detrimental changes than transitions (Lyons and Lauring, 2017). We chose three SARS-CoV-2 next generation sequencing datasets submitted by one single submitter with *n* > 200 samples to estimate intra-host nucleotide diversity occurring in 397, 374, and 215 next generation sequencing samples from Australia, Spain, and United States populations, respectively, using aligned reads per sample against the SARS-CoV-2 reference genome. This calculation has been already validated to capture intra-host viral diversity, overcoming sequencing errors (Nelson and Hughes, 2015). In the three populations, average nucleotide diversity positively correlates with the number of Single Nucleotide Variants (SNVs) with viral frequencies over 10% (Spearman correlation, *r*-values from 0.24 to 0.44, *P* < 0.0001). The latter supports the existence of intra-host minor variants and therefore SARS-CoV-2 quasi-species, coexisting within the same host (Miralles et al., 1999; Wright et al., 2011; Domingo et al., 2012; Ni et al., 2016); Figure 1H). As previously described, we hypothesized SARS-CoV-2 normally evolve by the action of APOBEC3G-mediating RNA editing (C > U) (Di Giorgio et al., 2020) including G > U and A > G changes in less extent, exerted by guanine-to-oxoguanine ROS-mediated generation and ADAR editing, respectively (Mourier et al., 2020). Conversely, hypermutants are mainly fueled by a higher intra-host diversity and homoplasies (different viral lineages emerged after the infection), reflected in more transversion changes that probably are maintained at low frequency in most SARS-CoV-2 infections, due the virus error correction machinery (Figure 1I). Taking together, and in agreement with others (van Dorp et al., 2020), we propose that the human host’s immune system substantially contributes to shaping SARS-CoV-2 genetic diversity, as evidenced in three distant population cohorts. Although intra-host diversity is probably one of the main sources of SARS-CoV-2 evolution, this is accompanied by RNA-editing at different levels, with SARS-CoV-2 RNA hypermutation as an extreme case of the latter, occurring in less than 2% of COVID-19 patients. This mechanism is predicted to inactivate the virus and is likely caused by host defense mechanisms involving higher RNA-editing as C > T (C > U) transitions and increased transversions, as frequent signatures observed in hypermutant genomes.

### Non-neutral Amino Acid Changes Actively Shape Evolution of Several SARS-CoV-2 Proteins

We next analyzed all inter-host major viral alleles occurring in SARS-CoV-2 genomes worldwide, by using the GISAID consensus called variants. 39,036 aggregated variants from GISAID genomes submitted until November 30, 2020 demonstrate that overall, A > G and T > C changes are the two most predominant nucleotide changes, over the referred C > U and G > U changes seen in NGS merged variants (see Figure 2A vs. Figure 1F). These changes were also present in GISAID samples with an outlier number of variants per genome, but A > G changes are not predominant in outlier samples, as seen with NGS outliers (Figure 2B). To deduce amino acid changes as consequences of these nucleotide changes, we analyzed nucleotide changes occurring in the aggregated GISAID variants and we predicted its consequences by using SnpEff (Cingolani et al., 2012), a program to annotate variants in VCF format available in the Galaxy server^15^. Occurrences per variant type demonstrate that missense and synonymous variant occurrences are more frequent compared to frameshift/non-sense variant occurrences per genome, and non-sense variants surpass frameshift variants (Figure 2C). Amino acid change analysis demonstrates frequent threonine (Thr > Ile), valine (Val > Ile), leucine (Leu > Phe) and alanine changes (Ala > Ser, Ala > Thr and Ala > Val), respectively (Figure 2D). Ala > Val, Thr > Ile and Leu > Phe changes are sustained by the C > U (C > T) transitions in the second position of the Thr and Ala codons, and the first position of the Leu codon, respectively. Val > Ile and Ala > Thr is caused by the A > G transition change in the first position of the valine codon and alanine codon, respectively. Other frequent changes, Lys > Asn and Glu > Asp are explained in part by G > C and/or G > T transversions. A priori, the previous nucleotides signatures are reflected in non-neutral SARS-CoV-2 amino acid changes that can affect SARS-CoV-2 protein structures and tend to be detrimental in terms of energetic changes, as previously demonstrated (Portelli et al., 2020). We performed several SARS-CoV-2 molecular dynamics simulations to see if these amino acid changes affect viral protein free energy trajectories. Two spike protein substitutions from European outbreaks containing Ala > Val (A222V, viral frequency = 17%) and Ser > Asn (S477N, viral frequency = 6.2%) including one Val > Phe substitution from a Brazilian outbreak (V1176F, viral frequency = 0.22%) readily changed Spike protein’s free energy-based motility to varying extents. The A222V change is predicted to decrease the motility of the N-terminal of Spike protein (NTD), while the S477N and V1176F variants are predicted to increase the motility of the Receptor Binding Domain (RBD) and Stalk domain of the Spike protein, respectively (Figure 2E). Previously, it has been shown that S477N slightly improves the folding of the Spike protein and the fitness of RBD-ACE2 binding (Starr et al., 2020) and more flexibility in the NTD could help to bind ACE2 receptor. Taken together, non-neutral amino acid changes in SARS-CoV-2 can change viral protein motility and might confer improved fitness to the virus, as appears to be the case of the S477N variant. In conclusion, we demonstrated a great diversity of changes occurring in SARS-CoV-2 with completely different outcomes in the Spike protein, as an example. Since many Spike protein variants are now being characterized in the laboratory with phenotypic characterization (Starr et al., 2020; Weisblum et al., 2020), it is important to integrate these studies with genomics data in real-time.

### SARS-CoV-2 3′ Untranslated Region (UTR) Is Evolving and Accumulating Greater Diversity

To gain insights into SARS-CoV-2 nucleotide variation in a population context, we divided SARS-CoV-2 genomes in 50 bp sequence bins and performed sliding window analysis to identify viral regions with skewing in viral frequency distribution toward low/rare frequency alleles using Tajima’s D statistic, a population genetics test to determine if these regions are evolving randomly or not (Tajima, 1989). Values of D that fell outside the middle 95% of the empirical distribution were considered potential outliers. Also, we calculated nucleotide diversity π in these bins and compared both values (see pi-tajima.sh script in out repository). Until November 30, 2020, the empirical distribution of Tajima’s D across Africa, Asia, Oceania, Europe, North America, and South America demonstrate consistent low nucleotide diversity in SARS-CoV-2 across bins, and negative Tajima’s D values (see black dots in Supplementary Figure 2). This is consistent with a viral population expansion and the inclusion of rare variants across SARS-CoV-2 genomes, as already reported (Liu et al., 2020). Outlying Tajima’s D values remain negative in all cases and bins 29,700 (region 29,650–29,700) and 29,750 (region 29,700–29,750) corresponding to the region ORF10-3′UTR are frequent outliers from the empirical distribution (smaller than the 2.5% percentile). Also, regions with high nucleotide diversity but not extreme values of Tajima’s D often span RNA-dependent RNA polymerase, Nucleocapsid and 3′–5′ exonuclease genes (see purple dots in Supplementary Figure 2). Overall, in every geographical region, bins containing most rare viral alleles outside 2.5% percentile of Tajima’s D values tend to accumulate toward 3′UTR of SARS-CoV-2 and not toward 5′UTR of the virus, specifically from ORF3a until the end of SARS-CoV-2 virus (Figure 3A). Consistent with the latter, ORF3a and 3′UTR regions are outliers (smaller than the 2.5% percentile) from a worldwide perspective. Of notice, bin 29750 (3′UTR), is an extreme outlier with a lower Tajima’s D value and higher nucleotide diversity compared with the rest of the bins (Figure 3B). The latter region corresponds to the highly conserved stem loop of SARS-CoV-2 (s2m, region: 29728-29768 Coronavirus 3′ stem loop II like-motif), conserved among coronavirus (Williams et al., 1999; Tengs and Jonassen, 2016) and essential for replication in other coronaviruses (Hsue et al., 2000; Goebel et al., 2004). Conversely, a bin corresponding with the 5′UTR of SARS-CoV-2 () present similar nucleotide diversity but not an extremely low Tajima’s D values as observed in the 3′ UTR regions (Figure 3B, purple dots). Overall, genes toward the 5′UTR in SARS-CoV-2 present the higher Tajima’s D values such as non-structural proteins nsp3, nsp4 the RNA-dependent RNA polymerase gene and the spike protein, among others. Interestingly, genes toward the 3′UTR of SARS-CoV-2 except for the E, M and ORF7b genes have the lowest Tajima’s D values, supporting that these genes are prone to accumulate rare viral alleles (Figure 3C). Until November 30, 2020 most of the non-sense variants in SARS-CoV-2 show variant accumulation in the ORF8 gene, as previously described, and suggest that ORF8 is dispensable for SARS-CoV-2 transmission (Pereira, 2020; Figure 3D). Regarding the latter, an outbreak in Singapore (45 genomes) contained a large 382-nucleotide deletion that truncated ORF7b and ablated ORF8 expression, but the transmission failed to continue (Su et al., 2020). The emergent SARS-CoV-2 B.1.1.7 lineage in United Kingdom contained at least 21 non-synonymous substitutions including a stop codon in the ORF8 gene (Q27^∗^, Figure 3E) and is constantly increasing its worldwide viral frequency in GISAID database since the beginning of November 30, 2020 until the time of writing of this manuscript (Claro et al., 2021; Galloway et al., 2021). Regardless the fact that all genomes from B.1.1.7 lineage contain the Q27^∗^ variant, around 14% of these genomes contain another downstream stop codon, Q68^∗^ (Figure 3E), confirming that ORF8 is prone to accumulate non-sense variants and B.1.1.7 lineage transmits successfully without expression of ORF8. ORF8 from SARS-CoV-2 has been shown to accumulate in the endoplasmic reticulum (ER) and activate an ER-mediated stress response that cause immune evasion via downregulation of the expression of interferon beta (Rashid et al., 2021) and the major histocompatibility complex I (Zhang Y. et al., 2020). Also, along with ORF3b, it is responsible for initiating an early antibody response in the host (Hachim et al., 2020). Thus, early neutralizing antibody responses in infections with SARS-CoV-2 B.1.1.7 lineage could be impaired (Sterlin et al., 2021) causing potential immune evasion (Neches et al., 2021). The population structure of lineage B.1.1.7 over time is similar to those observed worldwide with respect to Tajima’s D and π values. The genes that show the greatest diversity include the helicase and ORF8 genes and not those encoding for the nucleocapsid and Spike proteins. Notably, over time, the bin 29700 (ORF10-3′UTR) arose as an outlier among Tajima’s D values but not regions in the 5′UTR region of SARS-CoV-2 virus, as seen in the regional and worldwide population structure analysis of SARS-CoV-2 (Figure 3F).

The Tajima’s D-π combined graphs presented in this manuscript can be also useful to track the most diverse regions of SARS-CoV-2 and may challenge primer binding design strategies and test sensitivity (Osorio and Correia-Neves, 2020). We intersected worldwide Tajima’s D and π values against common primers used in qPCR testing. Among bins with Tajima’s D values lower than the 2.5% percentile of the empirical distribution, regions 28286–28306, 28308–28332, and 28334–28358 of SARS-CoV-2 intersect with CDC primers: 2019-nCoV_N1_Forward_Primer, 2019-nCoV_N1_Probe and 2019-nCoV_N1_Reverse_Primer, respectively (Supplementary Table 4). Since these regions are prone to accumulate rare alleles, it is possible that this fact can explain aspects of the false negative ratio of the SARS-CoV-2 test (Farkas et al., 2020; Khan and Cheung, 2020; Woloshin et al., 2020).

Taken together, several regions toward the 3′ region of the SARS-CoV-2 genome such as ORF3a, ORF8 and the 3′UTR (specifically in the s2m stem loop) but not the 5′UTR, contain an excess of low frequency variants relative to chance variation, evidenced by their outlying Tajima’s D values (Tajima, 1989). This distinction also applies to the ORF7a/ORF7b genes, where regions of sequence variation in ORF7a register the lowest Tajima’s D values, whereas these changes are not seen in ORF7b sequence. Thus, the Tajima’s D-π graphs can be helpful to identify and track these regions over time. Our pipeline offers a straightforward way to collect SARS-CoV-2 variants, consolidate them under the VCF format, and further apply downstream variant annotation and/or evolutionary analysis to identify regions under active evolution.

## Discussion

In this study we aimed to analyze over 230,000 SARS-CoV-2 sequences deposited between GISAID and SRA databases within the first 11 months of this pandemic (up until the end of November 2020) by using our pipeline. We characterized the existence of intra-host viral hypermutation that results in an excessive number of variants per genome that occurs in less than 2% of SARS-CoV-2 sequences (Figures 1A,B, respectively). This phenomenon was already described for HIV-1 virus *in vivo*, demonstrating that HIV-1 reverse transcriptase contributed only to 2% of mutations, and the majority was caused by host cytidine deaminases of the A3 family mediated editing (Cuevas et al., 2015). Since SARS-CoV-2 is subjected to this type of RNA edition (Di Giorgio et al., 2020), we propose that this enzymatic activity in combination with higher intra-host diversity contributes to SARS-CoV-2 overall diversity at a global level, leading to more than 39,000 major viral frequency variants within 229,000 GISAID genomes. In SARS-CoV-2 genomes, it has been proposed that the catalytic activity of APOBEC deaminases, adenosine deaminase acting on RNA proteins (ADAR), and reactive oxygen species (ROS), are the main drivers of SARS-CoV-2 variation (Mourier et al., 2020). The APOBEC-mediated C > T (C > U) transversion is substantially present both in hypermutants and non-hypermutant samples, suggesting APOBEC3G mediated RNA editing involvement, as previously reported in smaller sample sizes (Di Giorgio et al., 2020; Simmonds, 2020). Conversely, ADAR-mediated A > G transversion is not substantially present in hypermutant genomes (Figures 1E, 2A,B, respectively), arguing that ADAR-mediating RNA editing is not the main enzyme involved in the hypermutation mechanism. The hypermutated SARS-CoV-2 variant signature often contains transversions and non-sense variants that are predicted to inactivate several SARS-CoV-2 proteins, probably leading to an efficient mechanism of lethal mutagenesis to control viral spread (Figure 1F). Consistent with this, transversions are known to be more detrimental than transitions (Lyons and Lauring, 2017) and G > T and G > C transversions are predominant overall in the GISAID genomes. The first transversion has been already reported for other RNA viruses such as Maize streak virus (van der Walt et al., 2008) and has been linked with the formation of 8-oxoguanine, known to be the most common cause of spontaneous G > T (G > U) transversions in RNA (Li et al., 2006). The second transversion and the excess of other transversion changes in hypermutants can be explained in part by a guanine oxidation product, imidazolone (Kino and Sugiyama, 2001). Thus, it is possible that an exacerbated innate immune response followed by inflammation (Birra et al., 2020; Taefehshokr et al., 2020) can lead to hypermutation; nevertheless, according to this study, this response is extremely limited in the population at the RNA level.

Although we found significant inter-host variation in SARS-CoV-2, neutral evolutionary theory predicts most of these variants as having no or neutral effects (Gojobori et al., 1990). Most of the amino acid changes in SARS-CoV-2 have already been characterized as energetically detrimental (Portelli et al., 2020), and we agree with this fact in terms of structural dynamics, since recurrent changes in the Spike protein are capable of increasing (S477N, V1176F) or decreasing (A222V) the molecular dynamics of certain domains of the protein, implying that SARS-CoV-2 proteins are prone to evolve over time by variant accumulation. Nevertheless, variants with minimal changes in fitness such as the mutation D614G in the early months of the pandemic (Portelli et al., 2020) shifts the S protein conformation toward an ACE2-binding fusion competent state (Yurkovetskiy et al., 2020), thereby increasing infectivity (Plante et al., 2020; Zhang L. et al., 2020). Hence, it is difficult to predict real drivers of SARS-CoV-2 evolution using structural analysis alone but is important to continuously track these changes in order to integrate this data with the increasing knowledge of SARS-CoV-2 variation obtained in the laboratory. This is the case of ORF8 impaired expression in VOC genomes and the probable detrimental consequences on the early antibody responses in the host (Wang et al., 2020; Zhang Y. et al., 2020; Sterlin et al., 2021). Since stop codons in ORF8 are increasingly emerging, it is important to track these changes in the future and perform additional studies with these viral variants concerning their ability to elucidate a full immune response.

Population genetics can offer a view of how SARS-CoV-2 is evolving and rapidly characterize novel outbreaks. In this manuscript, we have proposed as previously done in the field of population genetics (Biswas and Akey, 2006) to computationally implement the calculation of Tajima’s D and π values across genome-wide scans of SARS-CoV-2 and estimate the empirical distribution of Tajima’s D values to dissect viral regions outside 95% percentiles of Tajima’s D. As proof of concept, until November 30, 2020 we observed an excess of rare viral alleles toward the 3′UTR of SARS-CoV-2, with the most extreme case in the two regions of viral sequence belonging to 3′UTR of the virus. One of these regions (29700–29750) falls into the last stem loop of SARS-CoV-2 (s2m, region: 29728–29768 Coronavirus 3′ stem loop II like-motif). It has been reported that s2m motif is highly conserved among coronavirus and unlikely to evolve due to this high degree of conservation (Williams et al., 1999; Tengs and Jonassen, 2016) and for its role in replication observed in other coronaviruses (Hsue et al., 2000; Goebel et al., 2004); nevertheless, at the beginning of the pandemic Australian genomes were reported to contain several variants in the s2m region, likely due to recombination events (Yeh and Contreras, 2020). We report that the accumulation of rare alleles in this region is frequently occurring worldwide and might represent two scenarios: this sequence was a recent acquisition in SARS-CoV-2 and is still in the adaptation phase within the host (Tengs et al., 2021); and it might represent a defense response exerted by the host, since this sequence has been demonstrated to be important in the viral replication process and proposed as a potential target for antivirals in SARS-CoV (Neuman et al., 2005; Robertson et al., 2005). Thus, it is important to track these changes as the pandemic continues to evolve.

In summary, we have presented potential molecular mechanisms that help researchers understand variation diversity fueled by natural selection in SARS-CoV-2, and we proposed a portable bioinformatic pipeline to collect viral variants, consolidate them as a single VCF format file and further calculate population genetic statistics to infer actively evolving SARS-CoV-2 regions. It is important to continuously track emergent viral variants with the bioinformatics tools developed and we hope these tools combined with others, can provide a bioinformatic platform for ongoing studies in SARS-CoV-2. We believe that this is an essential first step in identifying emergent forms of the virus but also underscore the need to perform structure-function based experiments of these variants using relevant preclinical *in vivo* models.

## Data Availability Statement

The original contributions presented in the study are included in the article/Supplementary Material, further inquiries can be directed to the corresponding author/s.

## Author Contributions

CF conceived of this study and performed all bioinformatics analysis and wrote the manuscript. AM performed mutant SARS-CoV-2 protein analysis and assist in biophysical studies. MT assisted in the statistical analysis, and data interpretation and manuscript writing. JH assisted study design, data interpretation and manuscript writing. All authors contributed to the article and approved the submitted version.

## Conflict of Interest

The authors declare that the research was conducted in the absence of any commercial or financial relationships that could be construed as a potential conflict of interest.

## Footnotes

1 https://coronavirus.jhu.edu/map.html

2 https://github.com/CDCgov/SARS-CoV-2_Sequencing

3 https://www.gisaid.org/

4 https://www.ncbi.nlm.nih.gov/sra

5 https://www.ncbi.nlm.nih.gov/sars-cov-2/

6 https://www.gisaid.org/

7 https://github.com/ncbi/sra-tools

8 https://github.com/ekg/freebayes

9 https://jacquard.readthedocs.io/en/v0.42/index.html

10 https://github.com/vcflib/vcflib

11 https://www.graphpad.com/scientific-software/prism/

12 http://software.broadinstitute.org/software/igv/home

13 https://instrain.readthedocs.io/en/latest/

14 http://manual.gromacs.org/documentation/

15 https://usegalaxy.org/

## Supplementary Material

Supplementary Figure 1 Benchmarking of detected variants between Pangolin and SARS-CoV-2-freebayes pipelines, using as input 229,162 GISAID FASTA genomes accessed until November 30, 2020. Venn diagram comparison between the Single Nucleotide Polymorphisms (SNPs) detected by the Pangolin pipeline (Rambaut et al., 2020) vs. SARS-CoV-2-freebayes pipeline. Both pipelines reconcile over 95% of detected SNPs from 229,162 GISAID genomes (see gray overlap). SARS-CoV-2-freebayes pipeline additionally detected Multi-Nucleotide polymorphisms (MNPs), Insertions, Deletions and Complex variants (Mixed) (see green circles, respectively). Number of variants are denoted with blue bold numbers.

Supplementary Figure 2 Empirical distribution of Tajima’s D values across geographical regions until November 30, 2020. Worldwide distribution of Tajima’s D values vs. nucleotide diversity (π) for bins of 50 bp in length derived from African (*n* = 4,301), Asian (*n* = 11,986), Oceanic (*n* = 17,211), North American (*n* = 47,658), South American (*n* = 2,325), and European (*n* = 145,884) GISAID genomes until November 30, 2020. The dashed lines correspond to the upper (97.5%) and lower (2.5%) percentiles of the empirical distribution of Tajima’s D for each bin. In both percentiles, genes containing the top three or four most extreme outlier bins are depicted with numbers. Also, genes containing the top three or four most diverse bins are depicted in purple. 3′UTR bins are depicted in red.

Supplementary TABLE 1 Sequencing metadata of 17560 Sequencing Read Archive (SRA) datasets downloaded until July 28, 2020. For every SRA dataset, we provided NCBI run accession, Assay type (indicates if amplicon, RNA-seq u other sequencing corresponds), sequencing size (bases, in nucleotides), Biosample accession ID, Center Name (depositor), release date, SRA study accession, BioProject and geographic location, among other information. The last column indicates the number of variants per sample (Major viral variants, viral frequency > 0.5).

Supplementary TABLE 2 Sequencing metadata of 229,162 GISAID genomes downloaded until November 30, 2020. For every GISAID genome, we provided GISAID genome name, GISAID unique identifier (Accession ID), geographic location, host, sequencing technology, lineage, and clade fields, among other information.

Supplementary TABLE 3 Acknowledgments from sequencing laboratories and/or consortia associated with GISAID genomes, listed in Supplementary Table 2.

Supplementary TABLE 4 Intersection between common primers for SARS-CoV-2 testing against bins outside 2.5th percentiles of Tajima’s D empirical distribution. Bins in the upper (97.5%) and lower (2.5%) percentiles of the empirical distribution of Tajima’s D from worldwide merged variants (*n* = 39,035) until November 30, 2020 were intersected against common primers for qPCR testing (as bed).

Supplementary File 1 Bioinformatic pipeline used for Molecular Dynamics Trajectory analysis implemented in this study.
