# Supplementary figures and images for: A Novel SARS-CoV-2 Viral Sequence Bioinformatic Pipeline Has Found Genetic Evidence That the Viral 3′ Untranslated Region (UTR) Is Evolving and Generating Increased Viral Diversity
Source: Front Microbiol. 2021 Jun 21;12:665041. doi: 10.3389/fmicb.2021.665041 (PMC8256173; doi:10.3389/fmicb.2021.665041)

Pangolin

SARS-CoV-2-  
freebayes

**SNPs**

**33445**

**1688**

**1169**

**224**

**Mixed**

**1854**

**Deletions**

**379**

**Insertions**

**1982**

**MNPs**

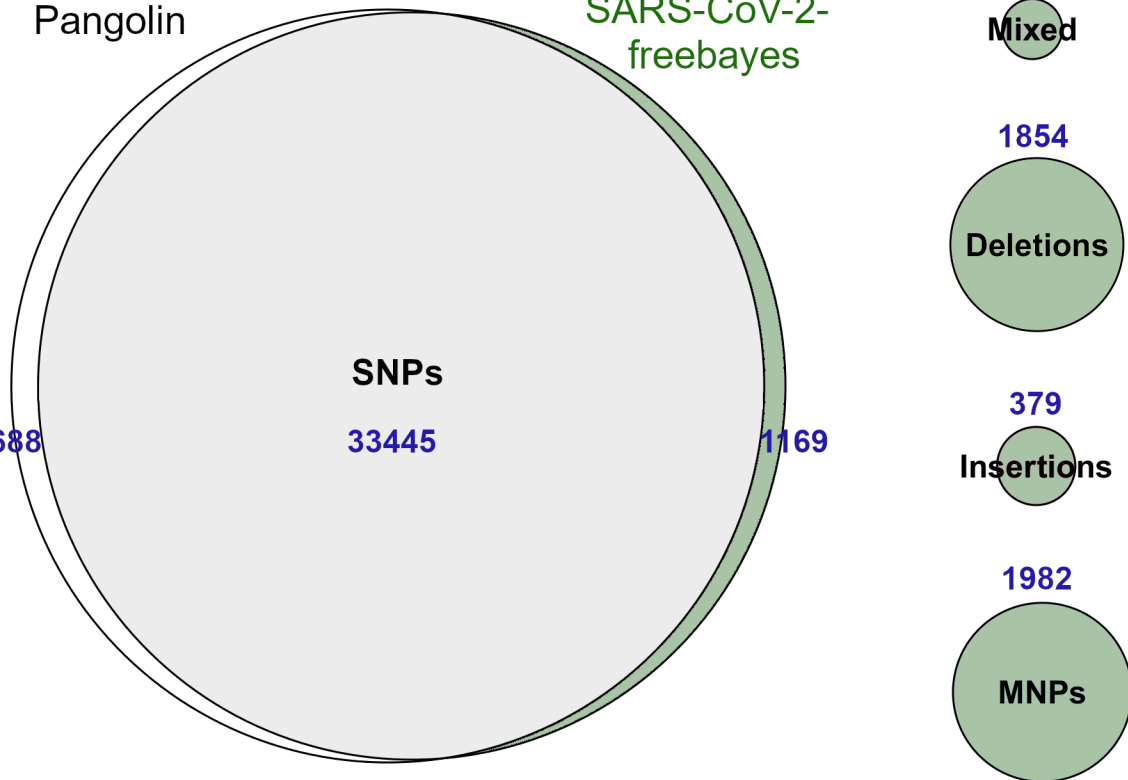

Supplement: Supplementary Figure 1 — Benchmarking of detected variants between Pangolin and SARS-CoV-2-freebayes pipelines, using as input 229,162 GISAID FASTA genomes accessed until November 30, 2020. Venn diagram comparison between the Single Nucleotide Polymorphisms (SNPs) detected by the Pangolin pipeline (Rambaut et al., 2020) vs. SARS-CoV-2-freebayes pipeline. Both pipelines reconcile over 95% of detected SNPs from 229,162 GISAID genomes (see gray overlap). SARS-CoV-2-freebayes pipeline additionally detected Multi-Nucleotide polymorphisms (MNPs), Insertions, Deletions and Complex variants (Mixed) (see green circles, respectively). Number of variants are denoted with blue bold numbers. [file Data_Sheet_1.PDF]

## Africa

n=4301

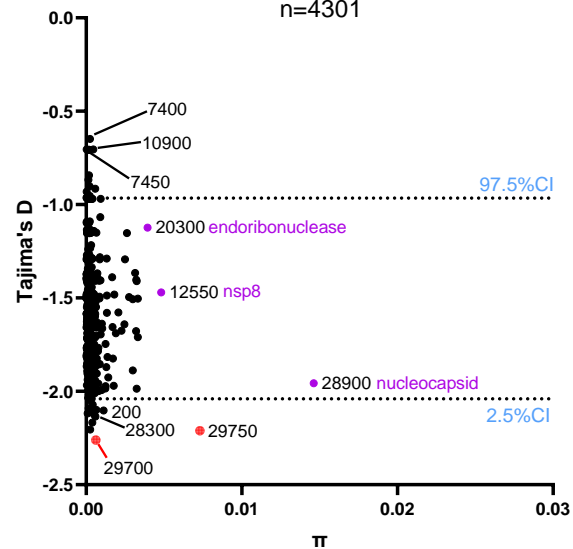

## Asia

n=11986

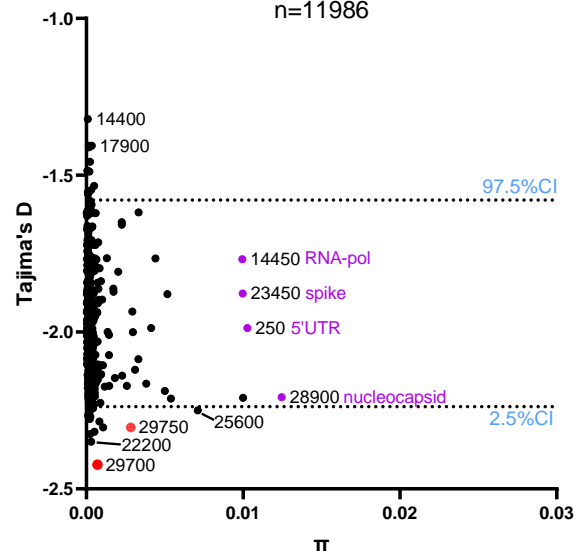

## Oceania

n=17211

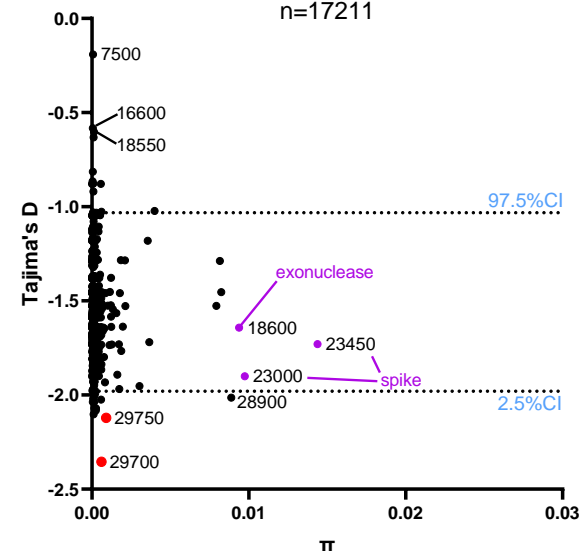

## North America

n=47658

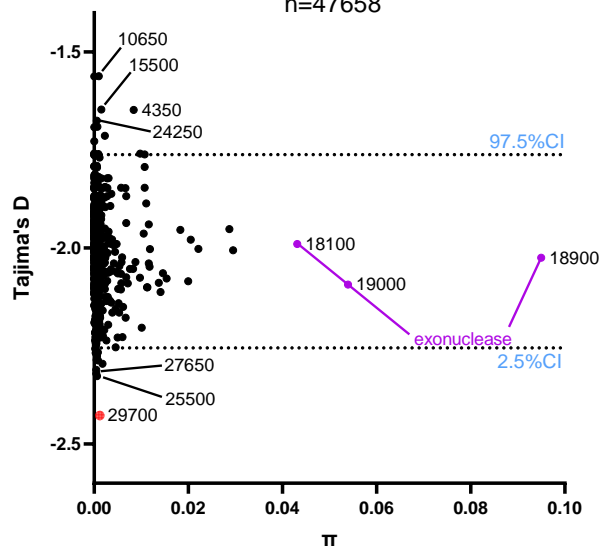

## South America

n=2325

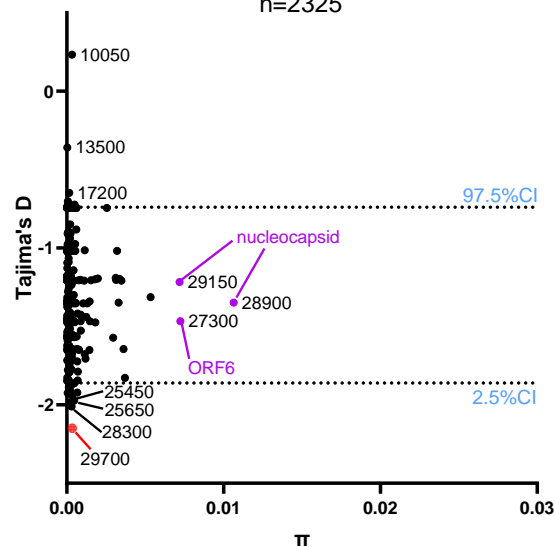

## Europe

n=145884

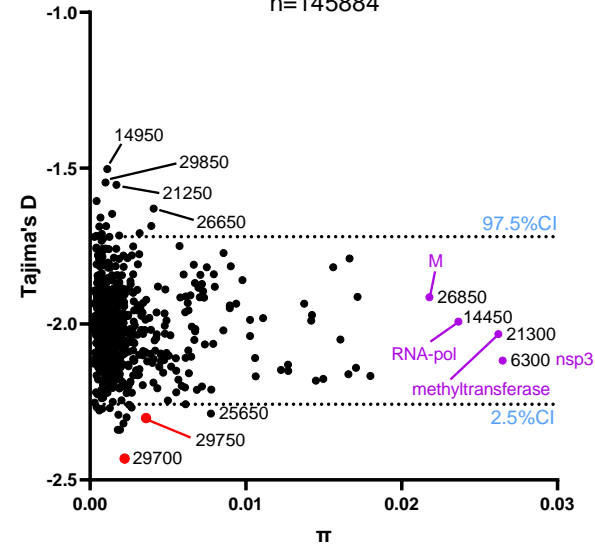

Supplement: Supplementary Figure 2 — Empirical distribution of Tajima’s D values across geographical regions until November 30, 2020. Worldwide distribution of Tajima’s D values vs. nucleotide diversity (π) for bins of 50 bp in length derived from African (n = 4,301), Asian (n = 11,986), Oceanic (n = 17,211), North American (n = 47,658), South American (n = 2,325), and European (n = 145,884) GISAID genomes until November 30, 2020. The dashed lines correspond to the upper (97.5%) and lower (2.5%) percentiles of the empirical distribution of Tajima’s D for each bin. In both percentiles, genes containing the top three or four most extreme outlier bins are depicted with numbers. Also, genes containing the top three or four most diverse bins are depicted in purple. 3′UTR bins are depicted in red. [file Data_Sheet_2.PDF]
