# Supplementary material for: A Novel SARS-CoV-2 Viral Sequence Bioinformatic Pipeline Has Found Genetic Evidence That the Viral 3′ Untranslated Region (UTR) Is Evolving and Generating Increased Viral Diversity
Source: Front Microbiol. 2021 Jun 21;12:665041. doi: 10.3389/fmicb.2021.665041 (PMC8256173; doi:10.3389/fmicb.2021.665041)
Supplement: Supplementary File 1 — Bioinformatic pipeline used for Molecular Dynamics Trajectory analysis implemented in this study. [file Table_4.DOCX]

**Molecular Dynamics Trajectory Analysis**

We submitted the jobs to the supercomputer infrastructure LEFTRARU NLHPC (ECM-02), allocating one node with a total 44 cores (logical) and one compatible GPU (NVIDIA Tesla V100-PCIE-16GB). We visualized the trajectories with VMD program version 1.9.3 (53). As example, for a given pdb (molecula_1.pdb), the commands used to perform the molecular dynamics are the following:

srun -p general gmx pdb2gmx -f molecula_1.pdb -o molecula_2.gro -water spce #1

srun -p general gmx editconf -f molecula_2.gro -o molecula_3.gro -c -d 1.0 -bt cubic #2

srun -p general gmx solvate -cp molecula_3.gro -cs spc216.gro -o molecula_4.gro -p topol.top #3

srun -p general gmx grompp -f ions.mdp -c molecula_4.gro -p topol.top -o ions.tpr #4

srun -p general gmx genion -s ions.tpr -o molecula_5.gro -p topol.top -pname NA -nname CL -neutral #5

gmx grompp -f 1.mdp -c molecula_5.gro -p topol.top -o em.tpr

gmx mdrun -nt 20 -nb gpu -deffnm em # EM

gmx grompp -f 2.mdp -c em.gro -r em.gro -p topol.top -o nvt.tpr

gmx mdrun -nt 20 -nb gpu -deffnm nvt # NPT

gmx grompp -f 3.mdp -c nvt.gro -r nvt.gro -t nvt.cpt -p topol.top -o npt.tpr

gmx mdrun -nt 20 -nb gpu -deffnm npt # NVT

gmx grompp -f 4.mdp -c npt.gro -t npt.cpt -p topol.top -o md_0_1.tpr

gmx mdrun -nt 20 -nb gpu -deffnm md_0_1 # MD

Where commands starting with “srun” (1-5) were executed directly in the cluster and the remaining steps were submitted via SLURM workload manager (<https://slurm.schedmd.com/documentation.html>). We choose forcefield OPLS-AA/L all-atom force field (2001 aminoacid dihedrals) for step one, and SOLVENT for step five (choice 13, SOL). We minimized atom clashes in the system with the steepest descent method (54), until potential energy were below 1000 kJ/(mol*nm). We considered a cutoff of 1.0 nm for non-bonded interactions under periodic boundary conditions (PBC). We performed NVT ensemble (constant Number of particles, Volume, and Temperature) setting no pressure coupling and modified Berendsen thermostat at 300K, respectively. We used in the NPT ensembles the Parrinello-Rahman barostat to keep the constant pressure at 1 bar, and the modified Berendsen thermostat to keep the temperature constant at 300 K, respectively. Long-range electrostatic forces were considered using the Particle Mesh Ewald for long-range electrostatics method (55). Both equilibrations were performed for 5000 picoseconds (5 nanoseconds). The total energy, temperature, pressure and the of the stalk domain trimmers were used to corroborate both system equilibrations. After these steps, production dynamics were carried out for 20 nanoseconds, by using the leap-frog algorithm with an integration step of 2 femtoseconds, as motion setting. We fixed bonds using the P-LINCS method, with constrained H-bonds (56, 57). Root mean square deviation (RMSD) and radius of gyration (Rg) were obtained with the following commands, respectively:

gmx rms -s md.tpr -f md_0_1.xtc -o rmsd.xvg -tu ns # Choose 4 ("Backbone") and Group 1 (Protein)

gmx gyrate -s md.tpr -f md_0_1.xtc -o gyrate.xvg # Group 1 (Protein)
